# SOCRAT: a Dynamic Web Toolbox for Interactive Data Processing, Analysis and Visualization

**DOI:** 10.3390/info13110547

**Published:** 2022-11-19

**Authors:** Alexandr A. Kalinin, Selvam Palanimalai, Junqi Zhu, Wenyi Wu, Nikhil Devraj, Chunchun Ye, Nellie Ponarul, Syed S. Husain, Ivo D. Dinov

**Affiliations:** 1 Statistics Online Computational Resource (SOCR), Department of Health Behavior and Biological Sciences, University of Michigan, Ann Arbor, MI 48104, USA; 2 Department of Computational Medicine and Bioinformatics, University of Michigan, Ann Arbor, MI 48104, USA; 3 Shenzhen Research Institute of Big Data, Shenzhen, Guangdong 518172, China; 4 Statistics Online Computational Resource (SOCR), Department of Statistics, University of California Los Angeles, Los Angeles, CA 90095, USA; 5 Michigan Institute for Data Science (MIDAS), University of Michigan, Ann Arbor, MI 48104, USA

**Keywords:** visual analytics, exploratory analysis, statistical visualization, web toolkits

## Abstract

Many systems for exploratory and visual data analytics require platform-dependent software installation, coding skills, and analytical expertise. The rapid advances in data-acquisition, web-based information, and communication and computation technologies promoted the explosive growth of online services and tools implementing novel solutions for interactive data exploration and visualization. However, web-based solutions for visual analytics remain scattered and relatively problem-specific. This leads to per-case re-implementations of common components, system architectures, and user interfaces, rather than focusing on innovation and building sophisticated applications for visual analytics. In this paper, we present the Statistics Online Computational Resource Analytical Toolbox (SOCRAT), a dynamic, flexible, and extensible web-based visual analytics framework. The SOCRAT platform is designed and implemented using multi-level modularity and declarative specifications. This enables easy integration of a number of components for data management, analysis, and visualization. SOCRAT benefits from the diverse landscape of existing in-browser solutions by combining them with flexible template modules into a unique, powerful, and feature-rich visual analytics toolbox. The platform integrates a number of independently developed tools for data import, display, storage, interactive visualization, statistical analysis, and machine learning. Various use cases demonstrate the unique features of SOCRAT for visual and statistical analysis of heterogeneous types of data.

## 1. Introduction

Contemporary decision making increasingly relies on data-driven evidence, advanced analytical methods, and artificial intelligence to extract valuable information from complex data and gain actionable knowledge [1]. Data visualization and analytics are crucial components of any study of large, heterogeneous, multisource, incomplete and time-varying data [2,3]. The goal of visual analytics (VA) is to support interactive graphical exploration, reasoning, and decision making with a combination of highly interactive visualizations and data science techniques [4]. This includes data management, computational transformation, hypothesis testing, and knowledge discovery. Graphical analytic workflows encompass iterative processes in which users motivated by specific questions interrogate datasets via interactive interfaces with support of dynamic visual and analytic components.

Many modern systems’ capabilities that provide exploratory data analytics (EDA) are either limited by requirements for platform-dependent installations of specific software (e.g., SAS, Excel, MATLAB, R, Python). These requirements are further coupled with necessary visualization expertise or specialized training that limits the utilization of advanced visual analytics methods. The rapid advances in massive data acquisition, web-based information, and communication and computation technologies support the explosive growth of interactive services and tools implementing novel solutions for web-based data exploration, visualization, and analysis [5,6]. Specifically, over the last decade, the information visualization community has witnessed the development of a number of general purpose systems and frameworks for online interactive visualizations, incorporating proven and efficient practices for enhanced compatibility, accessibility, and performance [5]. Web-based graphical solutions dramatically reduce deployment issues and improve portability by running directly in the desktop or mobile web browser. This approach yields significant scalability and high degree of accessibility, as well as avoidance of complex installation, version update demands, incompatibility problems, and other issues characteristic of standalone software deployment [7]. Together with increased graphical and interactive capabilities native to modern web browsers (supporting HTML5 format and active JavaScript), this enabled enhancement of visualizations by user-focused data-driven real-time interactions.

Furthermore, integrating statistical and machine learning methods with modern visualization components can greatly amplify the adoption and utility of visual data analysis [8]. Recently, improved computational capabilities of web browsers enabled implementations of mathematical, statistical, machine learning, and computing JavaScript libraries [6,9]. Combining these resources with existing interactive visualization frameworks can open a path to the development of more effective and powerful VA web-based systems without re-implementing standard components from scratch. However, there are a number of challenges associated with this endeavor, such as significant incongruences in design, development, and deployment. Earlier review of information visualization system architectures pointed out the difficulty of identifying common design patterns within existing visualization tools, and consequently the high cost for users to learn and evaluate unfamiliar systems [10]. Moreover, building general purpose visual analytics web systems is even more challenging than creating visualization tools, since VA application design requires their uniform integration with data management and analysis solutions into a large-scale web ecosystem composed of complex applications. Existing VA applications implementing this approach combine web-based interactive visualization libraries with specific analytical functionality, however, such solutions remain scattered and very problem-specific. Some solutions allow implementing web applications for visual data analytics in the form of web-hosted notebooks (e.g., R markdown and Jupyter electronic Notebooks) or dashboard-like tools (e.g., RShiny). These tools allow a developer to access a broad range of general-purpose analytical capabilities in corresponding programming languages (e.g., Python or R), and more recently, few high-level interactive visualization libraries (e.g., Altair [11]). However, the solutions implemented using these approaches are not native to web browsers, they require hosting with back-end capabilities and make integration of many other JavaScript data visualization and analytics packages not trivial. Thus, practices for development of sophisticated large-scale VA web applications are not yet well established [12].

Our motivation to address these challenges comes in part from decades of designing, building, and maintaining the Statistics Online Computational Resource (SOCR) [13]. SOCR includes a large collection of learning modules, instructional resources, EBooks, and web applications for in-browser data processing, analysis, and visualization. Despite the fact that this wide range of online tools is very popular among users, many of the applications were built as individual Java applets and separately re-implement common routines and data management operations. Such inefficiencies present the VA community with an exciting opportunity to pack some of routine data management, processing and analysis procedures into reusable components that could be used with well established information visualization solutions. Design principles satisfying these requirements are related to the best practices for large scale interactive front-end architecture optimization for more flexible, scalable, reusable, and sustainable web applications [12]. Lack of adoption and standardization of these techniques results in higher demands for development time and increased costs. Currently, more efforts are spent in re-designing VA system architectures and user interfaces, instead of focusing on interaction innovation and creation of sophisticated web VA applications. It is even more challenging for complex visual analytic solutions, where re-implementation of data processing, modeling, and analysis methods is very expensive and error-prone [14]. Given the advantages of the modern Internet, there is a compelling need for investing in building robust, extensible VA systems that can take advantage of a highly diverse landscape of visualization tools and analytical and computational web tools, but at the same time are easy to build and use.

In this work, we present the overall design and implementation for the Statistics Online Computational Resource Analytical Toolbox (SOCRAT). SOCRAT implements a modular web platform for building general-purpose holistic in-browser VA applications. We consider requirements for main steps of a general web-based visual analytics workflow and show SOCRAT data imports, displays, storage, interactive visualizations, and analytic functionalities. Instead of limiting the system by a fixed number of specific components, we provide a flexible and dynamic platform that enables easy integration of various data management, processing, analysis, and visualization modules. In order to achieve that, SOCRAT employs a multi-level modular architecture and declarative specifications that provide component independence and responsibility separation and enable easy customizations of the application, e.g., for a concrete project. We specifically focus on providing a convenient interface for simple integration of existing web-based tools for interactive data analysis and visualization. These abilities allow SOCRAT to balance expressive, interactive and processing capabilities, efficiency, compatibility, and accessibility. Along with system implementation details, we provide exemplary usage scenarios demonstrating applications of SOCRAT for visual analysis of real-life data. Lastly, we briefly discuss ongoing work, prospective developments, and potential community contributions.

## 2. Background and Related Work

Despite the rapid growth of applications for data analytics and visualization on the web, most of these tools are task-specific and are difficult to use together and there are few solutions that aim to provide a holistic, integrative platform to address these limitations. SOCRAT extends prior work on other web applications that provide enhanced interactive visualizations with data analytical capabilities, and specifically a large suite of previously developed 500+ SOCR tools.

### 2.1. Integrative web toolkits for visual analytics

Early web-based systems for visual data analysis relied upon Java applets and Adobe Flash to provide interactive graphics in the web browser. Improved graphical and interactive capabilities of modern web browsers promoted the development and wide adoption of open-source HTML5/JavaScript libraries, such as the commonly used D^3^ library [15]. These developments, however, are independent of implementations of computational and statistical JavaScript libraries that allow in-browser data processing and analysis. Often, the design of a particular visual analytics system focuses on a quite narrow area of application, together with a problem-specific approach to visualization, interaction, and analysis. There are few notable attempts to create integrative solutions that allow to efficiently combine existing and customly developed interactive visualizations and tools for data analysis.

WebCharts [16] is an integrative JavaScript framework that allows web applications hosting arbitrary visualizations. Although this approach does not directly provide any analytical capabilities, it makes integrative implementation of complex visual applications easier, since from both host application and visualization sides only support for WebCharts interface needs to be added to enable integration. Architecturally, SOCRAT is more similar to Plastic.js [17], a data display modular framework designed for extensibility, that implements asynchronous data loading and display, default inner data format and schema. As an integrative framework it supports usage of multiple visualizations solutions, including D^3^, and facilitates their dependent management and lazy loading.

Among solutions that provide out-of-the-box visual analytics, Tableau (formerly Polaris) [18] is a commercial framework, whose broad visualization capabilities are enhanced by data analytical methods, including data transformations and cleaning, statistical analysis, and clustering. Although Tableau allows embedding of created visualization into arbitrary web pages and provides API to interact with them, it is conceptually closer to VA toolkit, than a platform. Its web version, Tableau Cloud (formerly Tableau Online), implements most capabilities of the desktop application, but still misses some analytical techniques such as clustering and forecasting.

Even though Wrangler [19,20] does not provide visualization and analytical capabilities, it addresses the needs for interactive data wrangling or cleaning, one of the key challenges in data analytics [21,22]. Wrangler supports an iterative process with a spreadsheet-style interface that enables composing data wrangling sequences by manipulating a sample of the data in a web browser and applying these sequences to the full dataset.

Finally, the Vega ecosystem [23] includes multiple components implemented as Node.js packages in CommonJS format [24] that can serve as building blocks for developing in-browser visual applications. For example, implementation of the mixed-initiative for breadth-oriented EDA Voyager system and Tableau-inspired view specification tool Polestar rely on a number of separately released tools [25,26]. Among these modules are declarative visualization grammars Vega [27] and Vega-Lite [28], visualization recommendation engine Compass [29], and data utility library Datalib [30]. These components facilitate data loading and simple analytic capabilities including data type inference, summary statistic calculation, and string template matching. They can be easily combined, reused, and integrated into interactive applications, along with the modular D^3^ library starting from version v4 [15].

Limitations of existing tools open an exciting opportunity for creating web-based VA systems that benefit from bringing together modern web browser capabilities and best practices for building complex interactive web applications. SOCRAT is not only inspired by these projects, but in fact it allows their utilization, modification, and extension internally as modules, as described further in Sections 3 and 4.

### 2.2. SOCR tools for visual analytics

The design and development of SOCRAT heavily relies on our previous experiences from developing SOCR web applications [13,31,32]. SOCR implemented a collection of Java tools for in-browser interactive data visualization, modeling, and statistical analysis. Visualization components SOCR Charts [13] and Motion Charts [33] were based on open-source Java charting library JFreeChart [34] and allowed computation of data summary statistics and provided a number data representation types, including raw data display, data mapping, and over 30 various highly interactive data plots, charts and diagrams, including 3D, spatial, and cartographic visualizations. The SOCR Modeler [13] implemented interactive visual model fitting, including distribution mixture modeling and generalized expectation maximization implemented in the setting of 2D point clustering and classification. The SOCR Analyses component [32] provided hypothesis testing of both parametric and non-parametric models, data modeling (linear regression and ANOVA), and computation of power and sample size. SOCR Analyses was implemented using MVC pattern, allowing to decouple interactive visual representation from modeling techniques, such that the latter could be used separately as external computational library and easily extended. The SOCR Distributome project [35,36] addressed complementary computational modeling applications from the viewpoint of probability distributions. It includes tools for simulation, analysis and inference, model-fitting, examination of the analytical and computational properties of specific distributions, and exploration of the inter-distributional relations.

Originally designed for primarily educational purposes [37–39], the suite of SOCR tools has been proven over time to successfully realize visual analytics workflows, in which a user motivated by a specific analytical question interrogates a custom dataset via interactive interfaces, visualization displays, and data analytic components. Examples implementing such VA workflow include California ozone pollution case study using SOCR Charts and Analysis [40] and visual analysis of big medicare, labor, census and econometric data with interactive SOCR Data Dashboard [41].

Globally, since 2002, there have been over 18 million daily-unique users of the various SOCR services. The utilization of SOCR resources is split between academic institutions (e.g., instructors, courses, students) and non-academic users (e.g., industry, government and nonprofit sectors). The original SOCR Java applets were open-source and generally scalable, but did not build on a common platform to enable component interoperability, resource sharing, and runtime interaction. SOCR infrastructure realizes a suite of web tools providing many features important for VA workflows, but became disconnected and hard to maintain due to the lack of common infrastructure. Moreover, most of SOCR applets along with other in-browser Java based visualization tools became unsupported by major web browsers. Java and Flash browser support is mostly eliminated, which restricts the ability to embed Flash, Silverlight, Java and other plugin based technologies, in part as a response to numerous vulnerability reports [42,43].

This experience shows limitations of our earlier designs and implementations that inhibited our efforts to further expand and maintain them. We recently introduced preliminary design and development requirements to address some of these low-level structural limitations by using a modular architecture that optimized module interaction, re-usage, and extension [44]. This design broke down the functional core of an integrated VA system into atomic functional parts and proposed a way to compose them such that the whole system is flexible, extensible, and robust. However, the resulting design and module specifications were very low-level and verbose, such that specifying common components and their structures could be time-consuming and require dozens of lines of code.

In this work, we extend previous core platform design requirements to introduce a set of motivating design considerations, core component specifications, and user interface design. New design considerations update previous definitions by not only unifying various routine operations across different components of the system, but also by providing high-level specification protocols for various types of modules, including the support for the easy third-party software integration. In this version of SOCRAT, a configuration of a basic module typically requires fewer than 10 lines of code, and allows defining various characteristics of the module by the declarative specification of defined properties. Contributions to the development of SOCRAT as a platform are presented in Section 3. As a web VA application, SOCRAT effectively combines a number of third-party and custom developed modules for visual data analysis and interactive visualization, as described in more detail in Section 4.

## 3. SOCRAT Design and Key Features

Given a wide range of VA tasks that could be performed using SOCR and other web-based tools, we propose a new design of a flexible, dynamic, and feature-rich VA environment. The platform aims to provide a structural architecture reducing implementation efforts, yet supporting quick realizations of a wide range of visual analytics. The SOCRAT design promotes increased code reuse through a modular architecture and automatic wrapping of third-party solutions, while giving a developer an ability to integrate custom module functionality and supporting the robustness of the whole application in the runtime.

### 3.1. Design considerations

A basic requirements analysis for developing SOCRAT led to a set of considerations (**C**’s) based on previously developed platform design requirements [44]. Here, we extend them to address higher-level issues concerning the wide range of needs for visual analytics tasks, building an infrastructure from available tools, and customization of solutions.

#### C1. Include basic components of a typical visual analytics workflow.

We loosely define a set of main components important for typical analytics workflow as following:

- *data management*, including data input and preprocessing, storage, representation, and querying;
- *interactive visualizations* for various data types, including univariate, multivariate, high-dimensional, hierarchical, longitudinal, and geospatial;
- *data analytics*, including inferential and descriptive statistics, with applications of machine learning and other modeling and computational techniques, supported by interactive visual interpretation of the analytical results, method properties, and algorithm visualizations.

#### C2. Allow seamless data flow between components and their interaction.

This consideration is a further logical extension of **C1**. Having a number of generally defined modular components for visualization and analytics is not enough for their efficient usage as a system. In order to fully realize VA workflow, infrastructure design should facilitate their seamless integration and interoperability, such that the effort required from a developer to include a new component or replace an existing one should be minimal. Similarly, effort required by an analyst for switching between these components should also be minimized.

#### C3. Avoid reimplementation of components, when possible.

This consideration builds on the idea that since SOCR tools provided many unique out-of-the-box solutions, but without interoperability and using obsolete web technologies, missing parts that would address these issues should be implemented by taking full advantage of existing modern web solutions. This should be considered for building each of the basic VA workflow components as well as mechanisms for their integration. However, if existing tools do not include some previously realized unique features, their reimplementation should contribute them to be included in a toolbox.

#### C4. Allow flexible, complementary combinations of best tools and practices.

While it is possible to choose narrow-focused approaches, tools and data types for different goals while performing visual data analyses, it is possible to combine various best practices and tools to enrich SOCRAT feature set and provide an analyst with a wider range of tools to choose from. Possible issue with sometimes unnecessary and distracting abundance of tools is easily resolved by the ability to easily customize the application such that it is possible to guide an end user though a specific analysis protocol. This requires component independence, responsibility separation, and performance optimization.

#### C5. Extend basic components with available novel solutions.

This consideration takes into account the explosive growth of new tool implementations in fast developing fields of visual analytics, information visualization, and interactive web development. It is possible to implement basic VA workflow components defined in **C1** using only commonly used solutions that do realize current best practices as described in **C4**, however, early adoption of new techniques and their re-usage in SOCRAT benefits both developers and analysts. Tool developers receive an opportunity to test their implementation as a part of integrative infrastructure and SOCRAT users get early access to novel methods (see Discussion). Overall, an ability to quickly integrate open access components into common infrastructure further accelerates the development of the field and knowledge dissemination.

### 3.2. User interface

**Figure 1.**
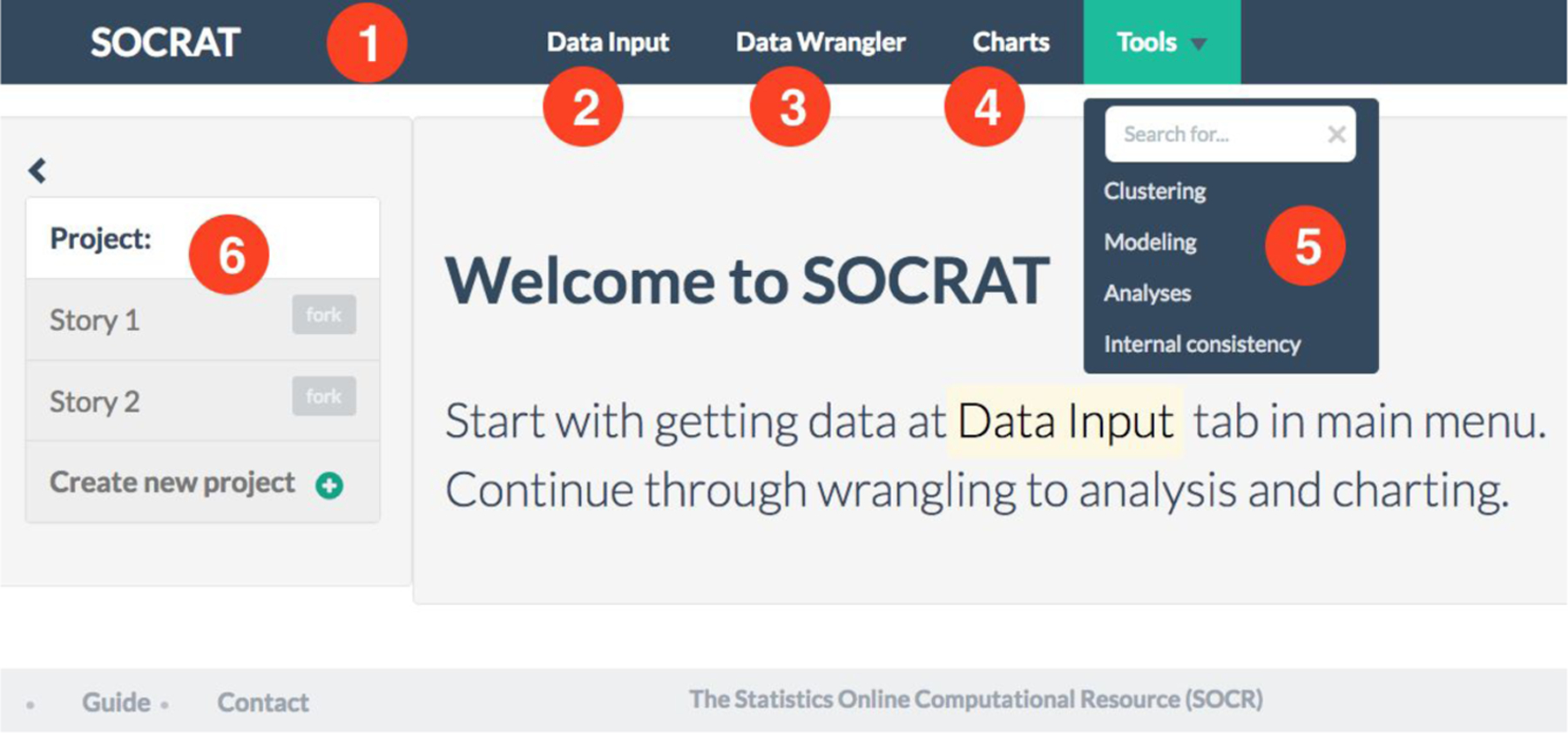
SOCRAT UI implementing an example of a high-level general VA workflow: **(1)** Main menu, automatically generated from the declarative configuration with active modules, including **(2)** Data Input module for loading and entering data; **(3)** Data Wrangler module for cleaning and transforming raw data; **(4)** Charts module for creating interactive visualizations; **(5)** Tools sub-menu for visually supported data analyses, including clustering, modeling and analytical tools; **(6)** Project management capability that will allow take snapshots of data, save visualizations and report analysis results.

**Figure A1.**
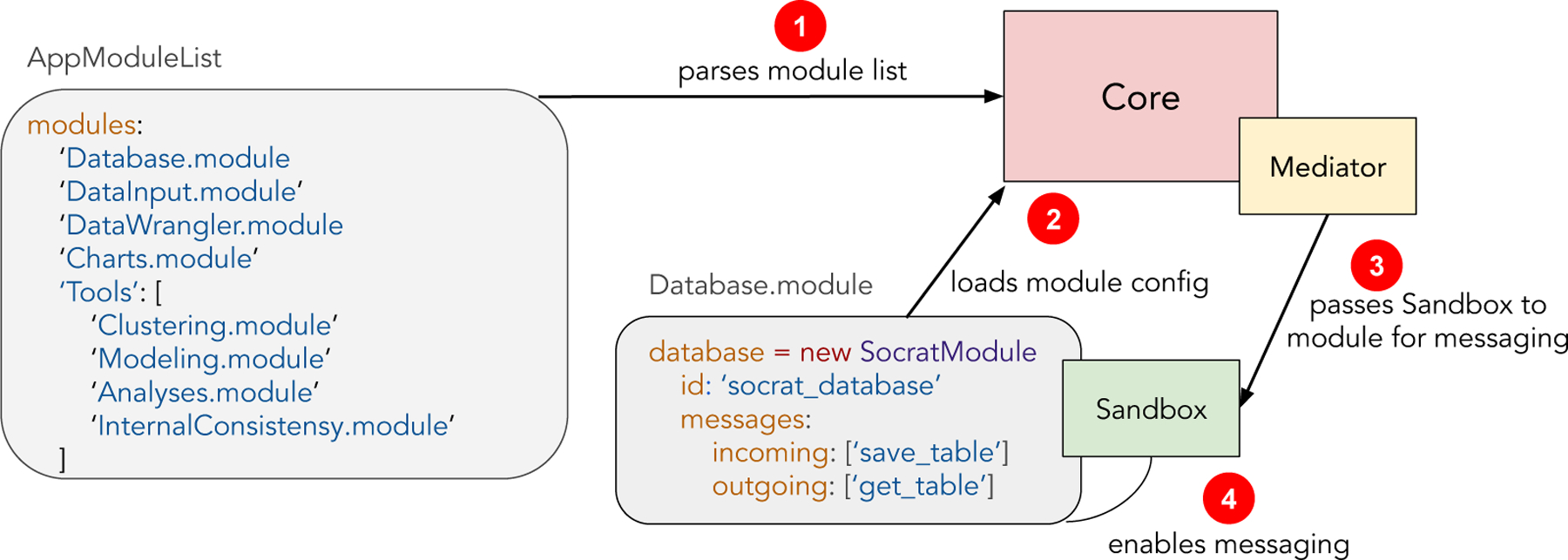
An example of a basic SOCRAT UI-less Database module configuration and initialization: **(1)** Core component parses the module list containing a link to the Database module config file; **(2** Core loads module configuration that implements an instance of a SOCRAT module with a unique module identifier; **(3)** Core invokes an initialization method of the Database module and passes an instance of the Sandbox component that serves as a proxy to access pub-sub functionality of the Mediator; **(4)** Using Sandbox, the Database module passes a list of messages that it can send out or listen to.

SOCRAT user interface (UI) consists of three top-level elements: main menu, sidebar and the central area. Figure 1 demonstrates an example of a SOCRAT application with UI modules representing general VA workflow steps (**C1**), from input and raw data representation, through wrangling, to interactive visualization and analysis. Main menu is used to provide access to central UI components and is defined declaratively via the active module list in JSON format that allows representation of hierarchical structures (Figure A1). Upon launching the app, this list is parsed recursively, initializing SOCRAT module configuration files that implement UI components (UI modules), which then are added into the main menu, while registering their corresponding URLs in the routing scheme. This takes the burden of constructing the main menu UI off the developer, while still allowing to organize it in many configurations, including multi-level nested sub-menus. When the group of modules defines a sub-menu (Figure 1), it will be automatically implemented as a dropdown component and will have a search field as the first item, that filters items by as-you-type live search.

A sidebar is a common UI pattern that is typically used for auxiliary purposes to the central page area. The only requirement for any SOCRAT UI module is to implement visual components and controllers for sidebar and main areas. UI components for both sidebar and main area are automatically initialized on the opening of every module’s URL registered in the routing scheme. A sidebar typically implements a request for the current dataset and allows manipulation of its properties, whereas the central area can be used as a view for particular visualization specifications. Once a UI module implements the sidebar and main area components, the developer is free to choose any visualization library with a combination of any analytical tools that can be further used inside this module.

Finally, it’s important to note that not all SOCRAT modules have to have a UI component. For instance, database that stores data values is a module, but it does not appear in the main menu. In the next subsection, we describe high-level specifications for different types of modules: a custom module with and without the UI, and an automatic wrapper module for an easy third-party component integration. From the technical perspective, we chose to implement SOCRAT using Node.js [45], AngularJs [46], and CoffeeScript [47]. These technologies are commonly used in the web data visualization and analysis community and provide a number of important capabilities discussed below.

### 3.3. Modular architecture

The main concept in the SOCRAT architecture is a *module*. While defining a rigid application structure with a number of modules with fixed roles would make their management and interoperability easier, it would drastically limit flexibility (**C4**), in the sense of application structure customization, module addition, removal or replacement. Moreover, tight coupling of the application components reduces their reusability (**C3**) and makes it more difficult to introduce changes to one without affecting the rest. Therefore, we designed SOCRAT modules as single-purpose parts of a system with limited permissions that do not directly communicate with each other. Instead, their interactions are supported via Core communication (**C2**). Core is one of the few basic SOCRAT system building blocks and is a singleton central control piece responsible for the initialization and internal communications of SOCRAT analysis modules. Core can start, stop, and restart individual modules selectively in the runtime, without disrupting the whole application, making the whole application more robust. Upon module initialization by the Core, each module is provided with a Sandbox, an interface to the Mediator, a publish-subscribe-like messaging service. This design reduces module interdependence, encapsulates module interaction, and promotes loose coupling, making the application more flexible, robust and scalable. Further, we first will consider specifications for modules with and without visual components or user interface. Then, we will describe the specification for intermodular communication, including the use of default and custom messages.

### 3.4. Basic module definition

Module structure is defined by the configuration file referenced in the master application module list (Figure A1). Basic module specification includes a single instance of a SocratModule class with one required field: a module id, which uniquely identifies the module in the system and is used for module initialization and intermodule communication. SocratModule class abstracts the logic of module initialization and serves as a wrapper on top of some important AngularJs functionality such as the dependency injection mechanism. When the application is started and the module specification file is parsed, the init method inherited from the standard SocratModule class is invoked. This method is used to provide the instance of the module with an instance of Sandbox that exposes the interface of the Mediator to the module. This interface allows the module to interact with the Core and, through it, other modules. The use of Sandbox reduces the possibility of a module interfering with the system by limiting its access to the inner mechanisms of the Core and Mediator. Through such implementation we also abstract a large amount of boilerplate code, allowing the developer to focus on creating independent modules in a few lines of code and connecting them with the messaging system that is described next.

**Figure 2.**
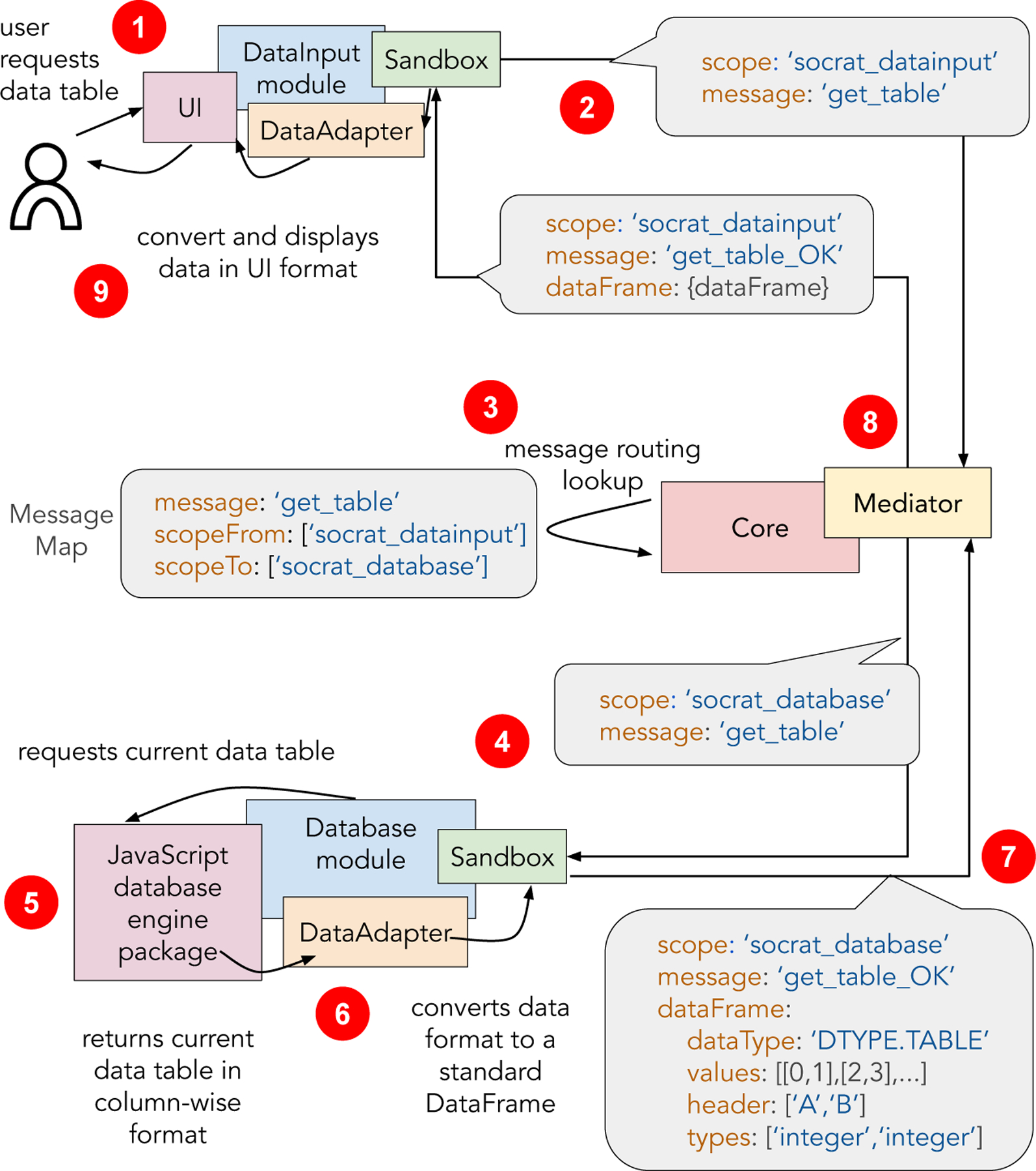
An exemplar scenario demonstrating SOCRAT module interaction mechanism: **(1)** The user analyst requests to view a current data table via DataInput module UI; **(2)** DataInput module sends out a message requesting the data table; **(3)** Mediator via Core uses MessageMap to look up modules that are listening to this message and automatically subscribes DataInput to the response message (with “OK” suffix); **(4)** Database module upon receiving message calls internal database storage to retrieve the current data table; **(5)** Database storage returns the data table in the internal format, e.g. column-wise; **(6)** DataAdapter component of the Database module implements data conversion to the standard row-oriented DataFrame format; **(7)** Database module sends the response with DataFrame object; **(8)** Mediator sends out a response message to the request initiating module; **(9)** DataInput module received the response and calls it’s own DataAdapter component to convert DataFrame into the format needed by the UI component to display the data table to the analyst.

As mentioned above, module communication in SOCRAT is implemented as a messaging interface (Figure 2). By default, each module automatically can use a number of standard messages defined globally, for example, for loading the current dataset into the module and saving it in the application. In addition, messages in the module specification can be used to define custom messages if needed (Figure A1). When modules are initialized, the Mediator automatically subscribes to listen to all outgoing messages from all modules. At the same time, module’s Sandbox subscribes to listen to incoming messages, providing a number of default services that implement callback methods for default messages. The developer still has to define callbacks for any custom incoming message. All messages go through the Core that ensures that there is at least one other module that is subscribed to the sent message and returns an error otherwise. This allows it to handle individual module failures and incompatibilities and fall back without breaking the whole application. Overall, this messaging mechanism is at the base of module interaction that enables data flow between different application components (**C2**).

### 3.5. Module interaction

The result of not imposing any higher-order structure on modules for the sake of flexibility (**C4**) is that the number of possible communication routes grows as *N*^2^, where *N* is a number of modules. In order to reduce complexity and improve maintainability of the implementation, Mediator acts as an intermediary for module interaction. Main advantage of Mediator is that it allows modules to broadcast or listen for messages without being concerned with the rest of the application. This means that messages can be handled by any number of modules simultaneously and adding features and their combinations can be scaled easily.

However, this approach raises two challenges. First, various custom modules can define their messages differently and when those have to be matched in order to pass information between modules. This is addressed via the message matching map, a central control point with a declarative specification that informs the Core of how intermodular custom messages should be wired (Figure 2). Another challenge associated with this approach is the possible difference in data formats that can be used by modules internally. For example, for loading and saving the current tabular dataset, the platform requires it to be stored in the specific DataFrame format, based on JSON schema. Each module is responsible for re-formatting their internal data representation into this common dataset format, which is validated by the Core when passing the message.

Figure 2 illustrates the module communication mechanism using an example scenario in which the user analyst requests to view the current tabular dataset in the UI component of the DataInput module. Sandbox, provided on initialization to the DataInput module automatically subscribed Mediator to all outgoing messages of this module, so now all DataInput has to do is to send one of those messages to make Mediator look up this message in the MessageMap. MessageMap defines in declarative fashion how Core should redirect messages from one module to another. Presence of this component is the price for loose module coupling that requires standardized interfaces in order to enable interoperability. To make message routing more compact, any message listed in the MessageMap file can share multiple scopes, i.e., it can be sent out and listened to by multiple modules simultaneously. This helps to avoid repetitive entries in the specification.

Another important step is data format unification. Since the design of SOCRAT aims to seamlessly connect various modules that can be using their own data types and formats (**C2**), it has to standardize those inner representations as well. To achieve that, SOCRAT defines a few global data types, for example, DTYPE.TABLE for tabular data. SOCRAT will attempt converting any JSON object to a tabular representation automatically and if that fails, it will use the DTYPE.NESTED type for an arbitrary data representation as a JSON object. It also provides the standard error service when the loaded module does not support the type of current data. The trade-off here is the need for each module to implement DataAdaptor, a component that will check current data types and convert data formats between internal representation and DataFrame. This may seem to be excessive, when the need is to connect a few JavaScript packages for visualization, but in practice this allows the developer to efficiently scale the system and to build feature-rich complex VA web applications that can efficiently handle dozens of dependencies. Finally, DataAdaptor code can also be reused between modules by abstracting it into a separate SOCRAT module.

### 3.6. Module specification with the user interface

**Figure A2.**
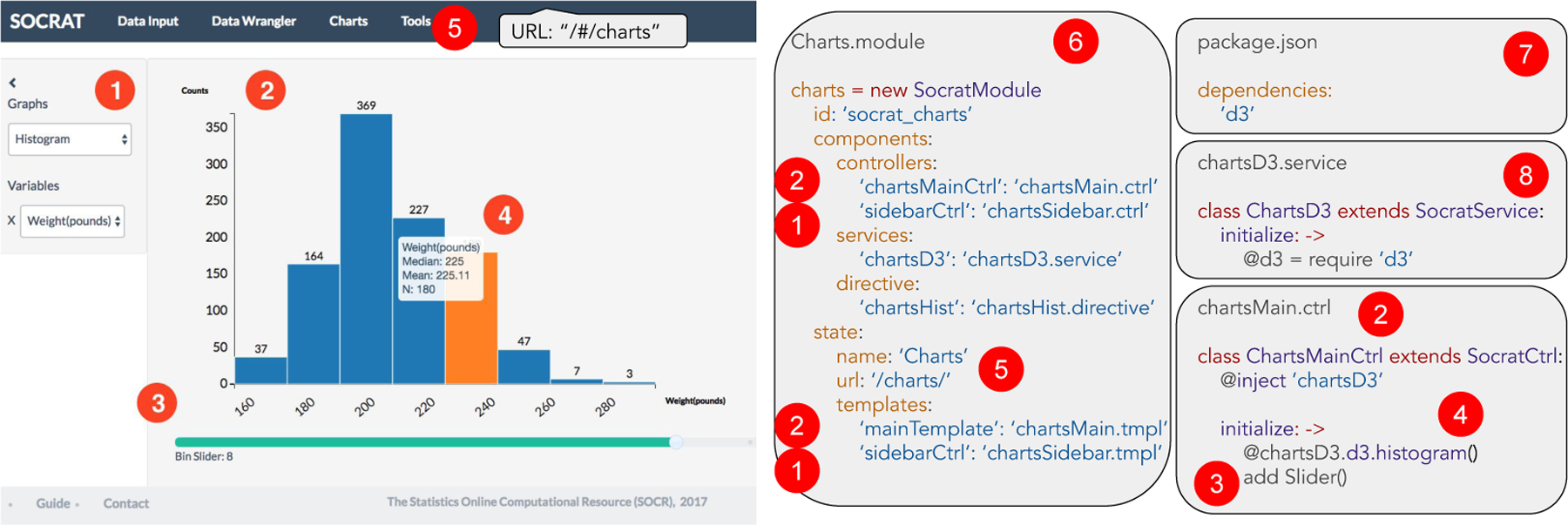
An example of the D^3^ chart implemented within the SOCRAT platform. Visual elements: **(1)** sidebar and **(2)** main area are two main UI components of any SOCRAT module; **(3)** additional control shows an interactive slider that allows to easily extend the standard D^3^ graphing capabilities within the application, **(4)** D^3^ histogram chart generated within the main area of the SOCRAT application. Corresponding SOCRAT module specification consists of **(6)** a config definition of the Charts module that includes **(5)** the module state, with url and main menu name components, as well as controllers and templates for **(1)** sidebar and **(2)** main area. D^3^ is also: **(7)** included as a global SOCRAT dependency and **(8)** injected directly into a service component.

Specification of a SOCRAT module with the user interface has another item state defined by name, url, and templates fields (Figure A2). These fields are used to build the visual component of the module (“view” in an MVC-type architecture) and integrate it into the application routing scheme. Available components include services, controllers, and directives, loosely following definition of AngularJS components. They are provided as SOCRAT base classes implementing special wrappers around AngularJS objects.

Upon module initialization, the platform will recognize the presence of the state item in the specification, which indicates that the module has a visual component. Module’s name is used to include the module into the dynamically created main menu of the application, while url is used for the routing, a typical mechanism of navigating the views in single-page web applications via modifications of the URL address without reloading the whole page. Fields mainTemplate and sidebarTemplate link to files with two major components of the module’s UI.

The main menu is used to provide access to SOCRAT modules that implement UI as per their state specifications (Figure 1). When a module is selected from the main menu, sidebar and main central areas are dynamically populated with the content from mainTemplate and sidebarTemplate, correspondingly (Figure A2). Two controllers connect the visual components (templates) with the business logic of the module implemented in services, thus completing an MVC-type architecture. Finally, directives are similar to those of AngularJS and implement custom elements with specific behavior.

### 3.7. Third-party module integration

The system architecture of SOCRAT allows for easy extension of system components (**C5**), for example, by adding support for third-party visualization or computational libraries. Extensibility is implemented in the form of wrapping third-party components as modules, providing them with a standard API for interoperability. We use the fact that many modern web libraries can be loaded as npm modules [48], exposing a single object with a simple high-level interface (e.g., D^3^ or Vega). While a third-party component can be made a dependency by composition (Figure A2(8)), abstracting it into a separate module allows better separation of responsibility (i.e., the rest of application does not depend on possible faults in the new component) and code reuse by other modules (**C3**).

To wrap third-party component, first it is added to the list of npm dependencies, such that the top-level object can be imported from the library using npm’s require statement when SOCRAT is configured and started. Then, the platform traverses the imported object’s interface and registers an incoming message for every found callable method. Now, it is possible to map another module’s outgoing message to such incoming message, such that when received it will result in an automatic invocation of the corresponding method of the imported third-party object.

To provide a specific example, Datavore [49] package is used for in-browser data storage with filtering and group-by aggregation querying capabilities. In order to start using Datavore as a SOCRAT module, it’s corresponding npm package has to be installed and listed as a dependency. Basic module wrapper with run-block needs to be implemented in order to initialize Datavore. Datavore package exposes a high-level object dv implementing data storage and querying API with a number of accessible methods. In order to make these methods accessible to the rest of SOCRAT, the wrapper will create a messaging interface that matches that of the Datavore object. Now, using Datavore from another SOCRAT module is as easy as sending a message to the Core. For example, a method for creating a new table dv.table() can be called by simply sending a message dv.table. At the same time, MessageMap allows to keep the rest of application independent of these technical details by defining a mapping between messages sent by other modules and data storage. Thus, replacing Datavore with an alternative data storage library with similar capabilities is easy and does not require changes in the rest of the application, besides MessageMap.

This example demonstrates how new tools can be easily integrates into SOCRAT as long as they are listed as npm modules. This allows for early adopting of components that are being actively developed, since as soon as new version of the package is listed as a dependency, SOCRAT will create new messaging interface and allow the rest of the application to access new features. At the same time, the rest of the system is decoupled from changes in the third-party package (except for MessageMap) and, therefore, is robust to possible malfunctions of this package. Integration by composition can still be useful, for example, when integrating a package with a specific data-UI model that is difficult to decompose and adapt to this scheme (**C4**).

## 4. Example Visual Analytics Capabilities

Given a wide range of VA tasks that can be performed using SOCR tools, we propose a number of various implementations employing popular web tools using modern web technologies. Per consideration **C1**, SOCRAT design defines three main components for visual analytics workflow, which are briefly discussed below. Examples in this section focus on the analysis of tabular data, the most commonly supported data type in visual analytics. However, SOCRAT can be used to build VA environments with other data types, as described in Section 3.5.

### 4.1. Data management tools

The first component is data management and it includes modules for data input, representation and storage, querying, and wrangling. Loading various datasets into a web VA system is an important first step in the majority of typical VA workflows. Many existing VA applications provide a few predefined demo datasets and also support uploading files. High diversity of data inputs not only provides an analyst with a bigger number of options for entering data, but also allows developers to distill best VA practices applicable across domains, dataset types and sizes, and perform better testing and more robust development of the tool. Similarly, most existing web VA solutions assume that input data is clean, correctly formatted, congruent, and homogeneous. While dataset wrangling or cleaning in general is a big challenge in data analytics and there is no system that addresses end-to-end iterative data cleaning process [19]. Providing an analyst with options for in-place wrangling can reduce the need to switch between a number of specialized tools, which motivates integration of such features in the SOCRAT design.

#### 4.1.1. Data input and display

The design of SOCRAT allows easily implementing a number of ways that an analyst can use for this purpose. It includes:

- Handsontable [50], a dynamic Excel-like data grid editor for raw data display and input via copy-and-paste;
- options to upload or drag-n-drop delimiter separated files such as CSV/TSV or JSON files, which SOCRAT will attempt to convert to tabular format;
- requests to various application program interfaces (APIs), e.g., World Bank APIs [51];
- over 50 predefined datasets are available from the SOCR Data resource [52], including climate, economic, business, and census datasets along with scientific data derived from multiple biomedical and healthcare studies.

**Figure 3.**
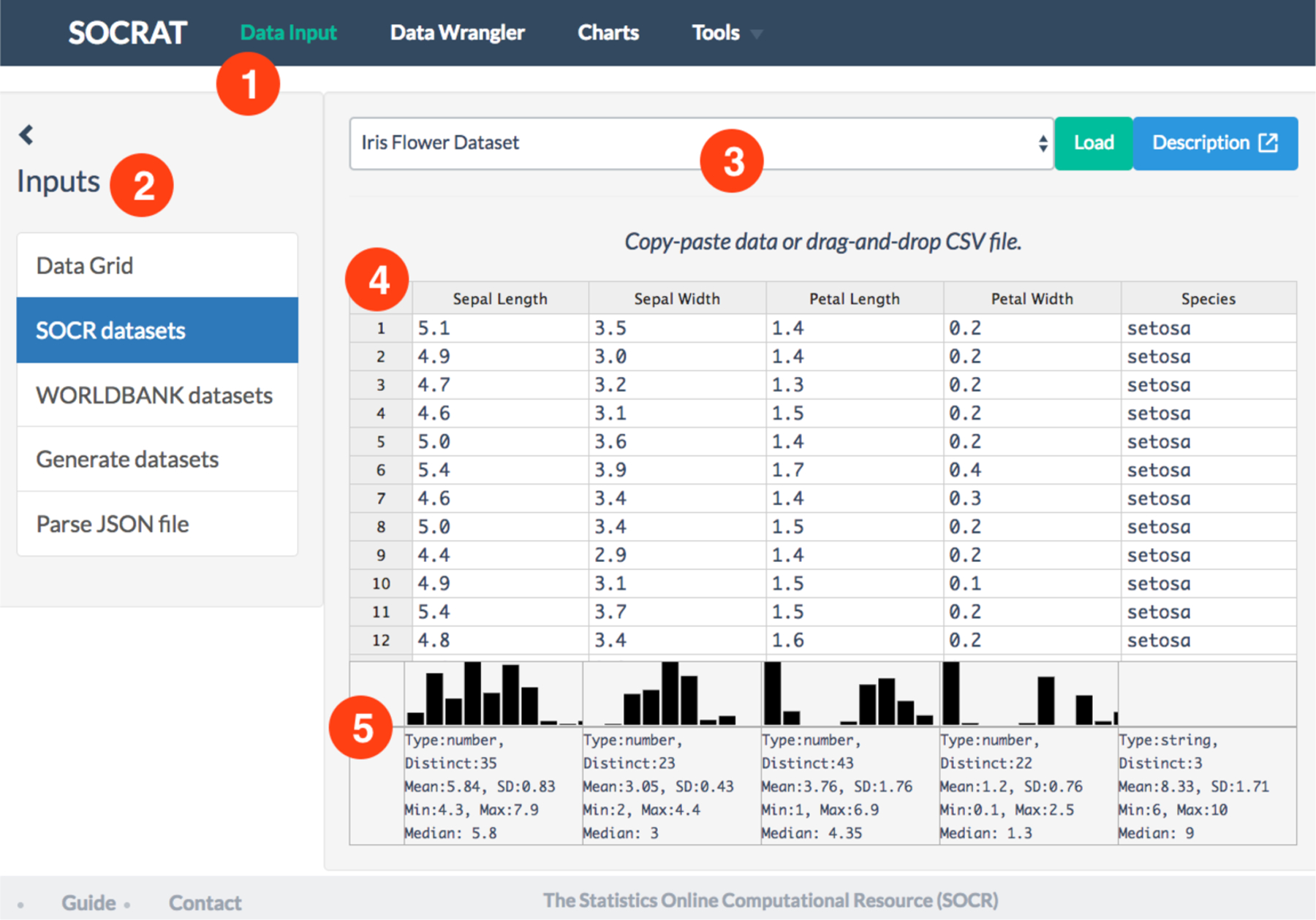
Overview of DataInput module: **(1)** user interface includes sidebar with various data sources and central area panels with data view; **(2)** data sources include data grid, a number of predefined SOCR datasets, ability to load data from the World Bank using web API; **(3)** central panel includes source-specific secondary controls, including links to dataset description; **(4)** dynamic, editable spreadsheet-like data grid contains raw data values view and also allows to drag-and-drop CSV/TSV file to load the data; and **(5)** summary information panel below the data grid shows histogram and reports summary statistics for for each variable in the famous Iris flower dataset.

As an example of the integration of a third-party component (**C4**), we implemented the DataInput module that utilizes the powerful online spreadsheet-like editor Handsontable [50]. Handsontable is used to display raw data values in a scrollable grid with dynamic loading of content, which allows the analyst to briefly glaze over the values in familiar Excel-like style. Additionally, we implemented a module that wraps third-party data utility library Datalib [30]. Upon data loading we use Datalib for tabular data parsing, column type inference, and summary statistic calculation, including histogram generation that is then rendered using D^3^. As a result, DataInput module can display per-column summary statistics and histogram of values above each corresponding column of the dataset to improve efficiency of initial data exploration, see Figure 3.

Despite the fact that Handsontable is capable of dynamic rendering of a comparatively large number of data cells, many downstream modules, for example, for data wrangling, processing, and analysis do not always scale that easily. Thus, we can use another component that provides a number of row subsampling strategies, including random uniform subsampling with an option for stratification. In current implementation row downsampling is offered automatically, when the total number of values in the current dataset exceeds 20,000 entries. As a result, we have a powerful implementation of the DataInput module that allows the user to enter data into the application in multiple ways and utilizes a number of popular JavaScript packages for data view and analysis, including Handsontable, Datalib, and D^3^.

#### 4.1.2. Data storage, querying, and wrangling

For data storage and querying SOCRAT can also reduce re-implementation of common functionality by utilizing third-party components. Next example for data storage and querying implements Database module that wraps Datavore [49], a small, fast in-browser JavaScript database engine. Datavore consists of an in-memory column-oriented database that enables fast filtering and group-by aggregation queries for data transformations. Datavore can complete queries over million-element data tables at interactive (sub-100ms) rates. Data is saved into Datavore after parsing and type inference and further can be used by other SOCRAT modules by sending request messages via the mediator which then SOCRAT core redirects to Database module, as shown in Figure 2.

**Figure 4.**
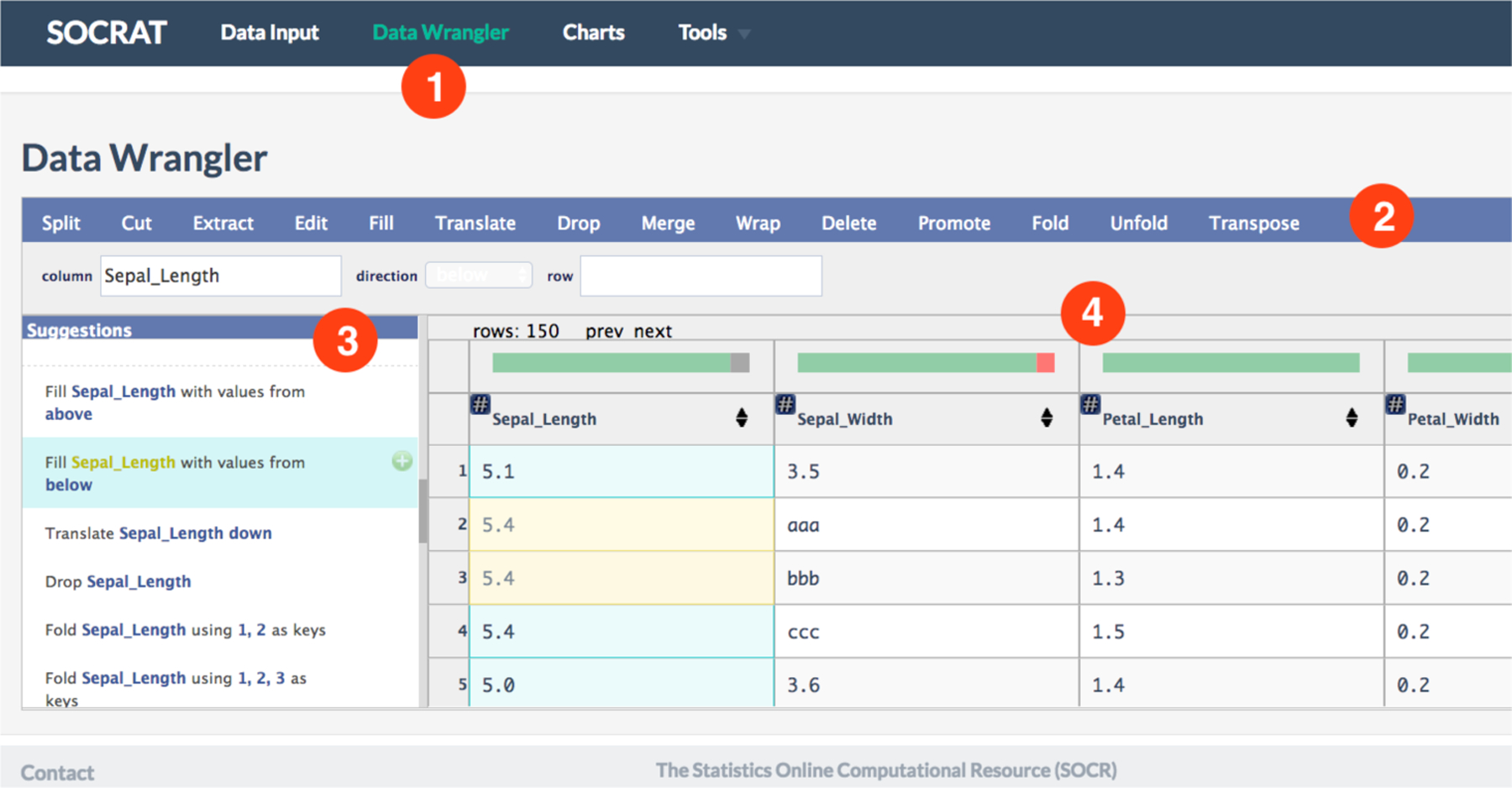
Data Wrangler module overview: **(1)** it features original Wrangler interface integrated into SOCRAT; **(2)** all original Wrangler operations are included; **(3)** Data Wrangler hides standard SOCRAT sidebar to free up space for Wrangler transformation suggestions panel; **(4)** Wrangler data diagnostic shows indicators of missing and erroneous values and inferred data types for dataset loaded from Database.

Wrangler [19,20] allows highly interactive in-browser data cleaning and transformation supported by analytics and visualizations. It couples a mixed-initiative user interface with an underlying declarative transformation language. We created a SOCRAT module for data wrangling that utilizes both Wrangler and Datavore. In order to do that, we modified Wrangler, originally released as a standalone web application, by transforming it into a standard Node.js module format [48] and adding it into SOCRAT as a dependency. We use npm to include and install system dependencies defined in a declarative way in the configuration file. Updates in Wrangler implementation included extension of limits on acceptable number of columns, updating Wrangler library dependencies, and other small bug-fixes. As a result, Wrangler is integrated by composition in a separate SOCRAT UI module and declaratively specified in the SOCRAT platform configuration to appear in the dynamically built main menu, see Figure 4. This allows the application to suggest to an analyst a number of applicable transforms on selected data, including automatically generated string extraction rules. Within SOCRAT, Wrangler requests and parses the current dataset from Database on start and sends the result back on exit, utilizing SOCRAT intermodule messaging interface.

### 4.2. Interactive visualizations

**Figure 5.**
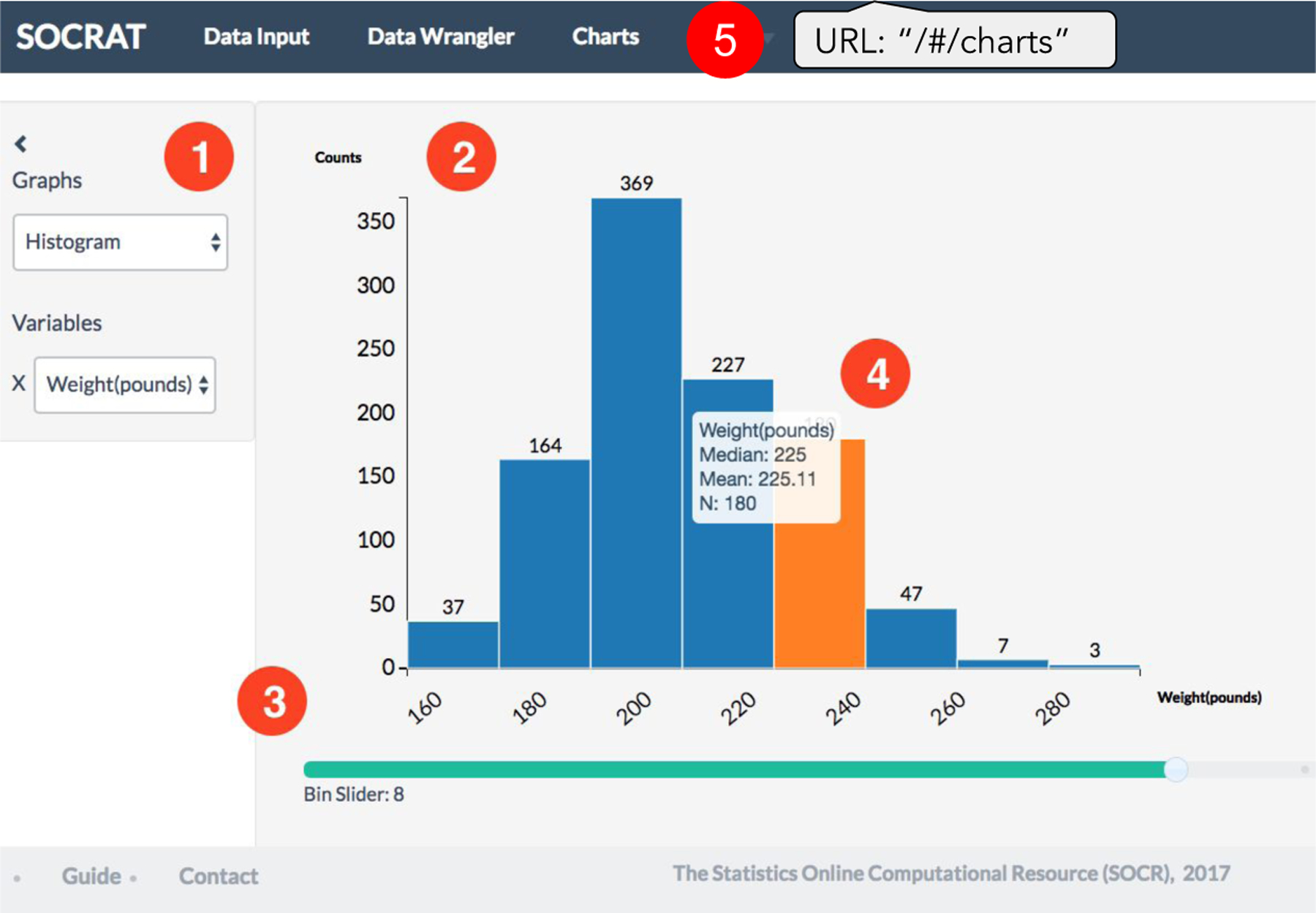
An example of the D^3^ chart implemented within the SOCRAT platform. Visual elements: **(1)** sidebar and **(2)** main area are two main UI components of any SOCRAT module; **(3)** additional control shows an interactive slider that allows to easily extend the standard D^3^ graphing capabilities within the application, **(4)** D^3^ histogram chart generated in the main area of **(5)** the Charts module.

Modern landscape of web-based interactive visualization tools is highly diverse. Ubiquitously used and well documented visualization framework D^3^ [15] provides declarative mapping data to visual elements on the web page and incorporates proven and efficient practices for improved compatibility, accessibility, and performance. Integration of D^3^ enables fine-grained control to create customized, specialized visualizations for various VA applications (Figure 5). However, the vast majority of the world’s visualizations instead are produced using end-user applications such as spreadsheets and business intelligence tools, which motivated the design and the development of higher-level declarative grammars for describing visualizations, for example, Vega and Vega-Lite [27,28]. While most existing VA applications make a choice and employ only one of these approaches, it is also possible to combine them (**C4**).

**Figure 6.**
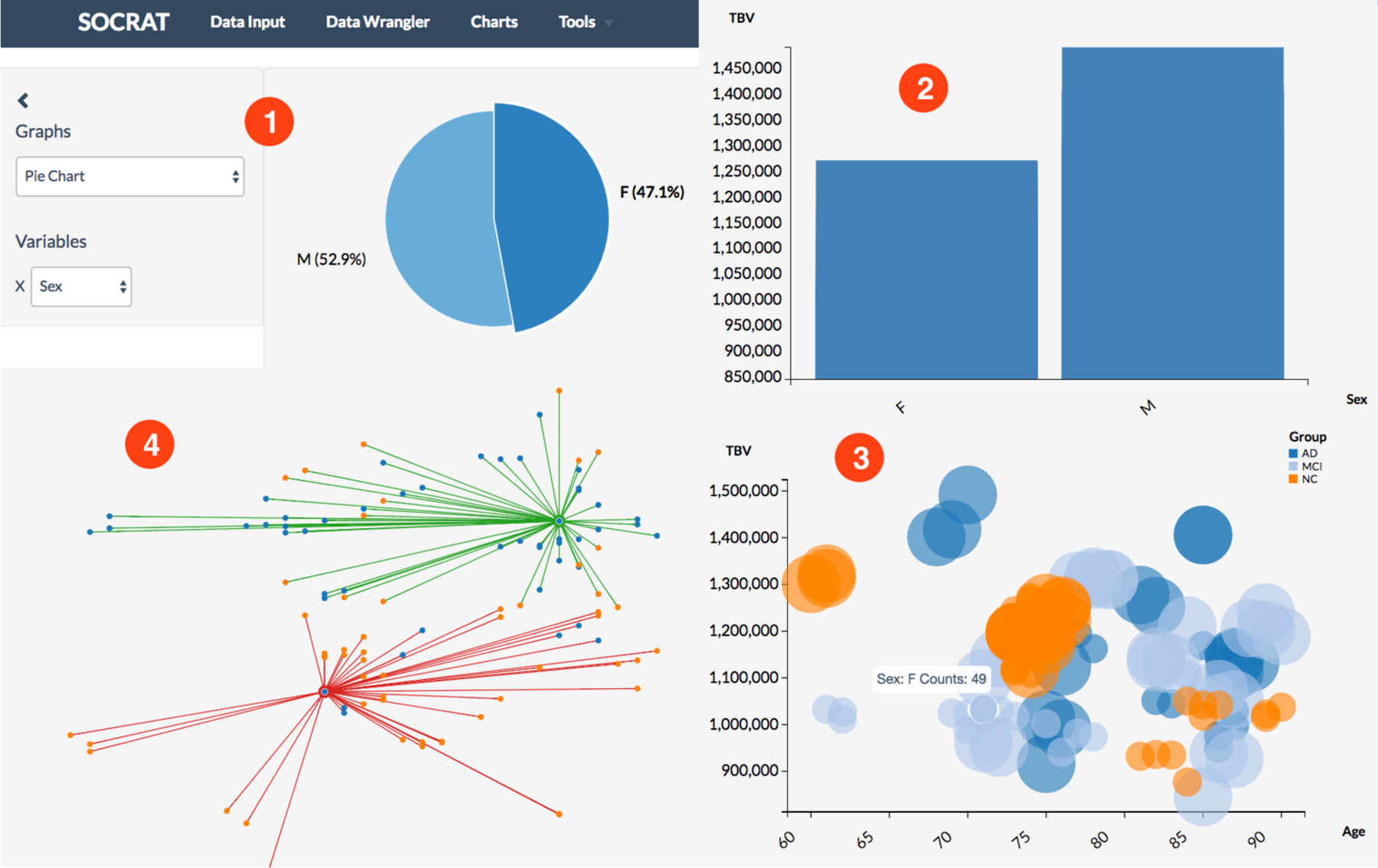
Exploratory visualizations of a SOCR dataset [52] obtained from a neuroimaging study of 27 Alzheimer’s disease (AD) subjects, 35 normal controls (NC), and 42 mild cognitive impairment subjects (MCI) [54]: **(1)** patient sex ratio; **(2)** total brain volume in males vs females; **(3)** relationship of total brain volume with age, sex, and diagnosis; **(4)** results of k-Means clustering total brain volume and age by sex (*k* = 2).

**Figure 7.**
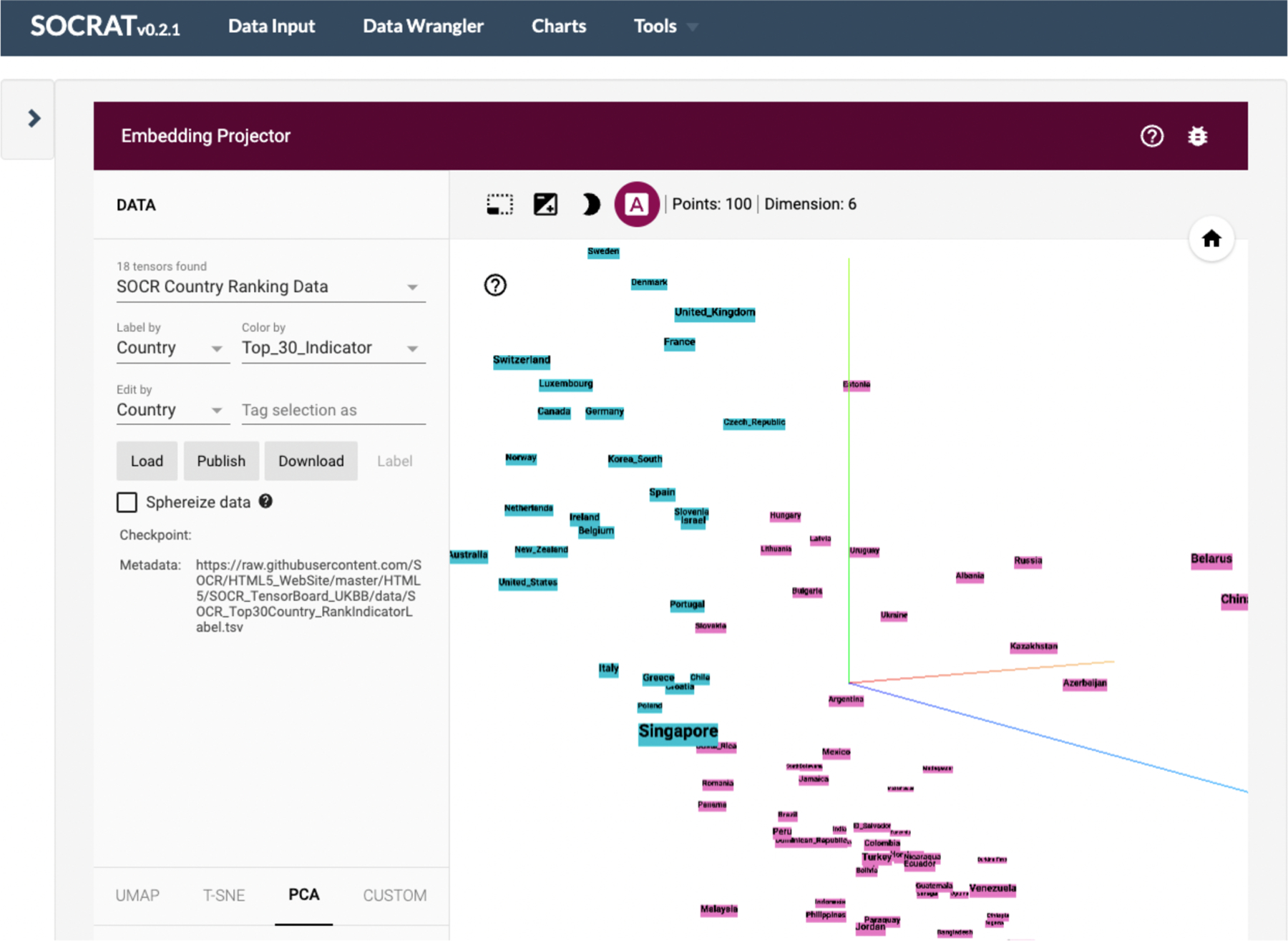
An example of the third-party interactive EDA solution running inside the SOCRAT plat-form on user-defined data. This module integrates the Embedding Projector [53] that demonstrates a dynamic 3D visualization of PCA decomposition of the SOCR country ranking dataset on political, economic, health, and quality-of-life factors [52].

Inclusion of multiple visualization frameworks into one platform presents with a challenge of instrument interoperability, responsibility separation, and inflation of the application deployment artifact size that could negatively affect the performance. However, modular implementation of tools within the Vega project [23] and new “microlibrary” approach used in D^3^ v4 fits well into loosely coupled SOCRAT core platform architecture. It prevents loading all of these visualization libraries in the global context of the application, and allows instead to use their subcomponents selectively, while loading them dynamically on-demand. By taking advantage of that fact and by employing decoupled modular architecture that minimizes component interdependencies and conflicts, SOCRAT enables best practices in web visualization (**C4**) by simultaneously using multiple different visualization frameworks. For example, its current version employs: (1) Vega-Lite [28], when high high-level declarative description is enough (Figure 6(1)), and (2) low-level D^3^[15] library, when detailed control over chart components is required (Figures 5 and 6(4)). Other visualization tools, for example, Embedding Projector [53] are also easy to integrate via module API-wrapping mechanisms described in the previous section, see Figure 7.

The ultimate goal of the implementation of the general visualization module in this example is to provide capabilities to replace some of the widely used Java-based SOCR charts. In order to achieve that we implement a SOCRAT charting module with dynamic input data reformatting in standardized way such that it can be easily consumed by typical visualization frameworks. All chart implementations are structured similarly and use the sidebar panel to specify detailed visual data encodings according to inferred data type. For instance, this design is similar to Polestar and Voyager systems [25], however, when fully transitioned, our charts module will provide over 30 various types of easily customizable chart configurations for in-depth analysis of multivariate tabular data based on histograms, scatter plots, line, area, bar, bubble, and pie charts (Figure 6). The Charts module also employs Datalib from the previous example to perform data type inference, which allows automatic support for time series data such as streamgraph. For exploration of hierarchical data represented by JSON objects our module implements treemap and graph charts.

### 4.3. Data modeling and analytics

**Figure 8.**
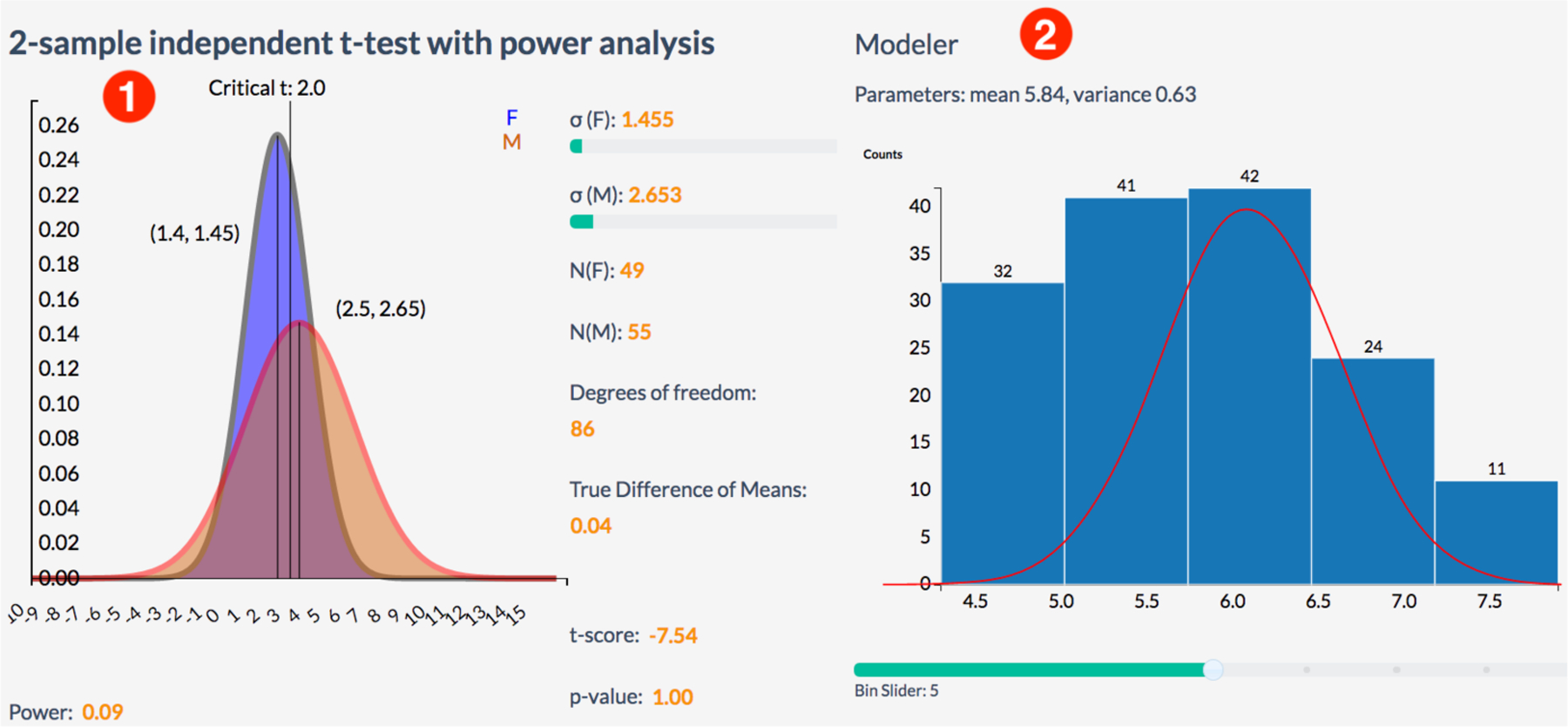
Examples of interactive tools for statistical analysis: **(1)** Analyses provide visually supported two-sample t-test with power analysis to compare mean of variable stratified by category (sex); **(2)** Modeler provides univariate normal distribution fitting that utilizes interactive histogram and overlapping line plot for fitted probability distribution and reports its parameters.

Third component covers methods for data analytics. Both graphical (exploratory) and confirmatory (explanatory) analytical methods are important for gaining knowledge from data and for decision support. Inferential statistical methods allow us to derive knowledge from data samples about population properties by testing hypotheses and deriving estimates of underlying probability distribution. Traditional methods for visual statistical analytics include data modeling and analysis using parametric and nonparametric techniques supported by graphical model diagnostics (Figure 8). Exploratory analytics, on the other hand, rely solely on the properties of the observed dataset and aim to summarize its main characteristics and discover patterns (Figure 7). Any of these methods can enhance and be enhanced by interactive visualizations for both more effective analysis and better understanding of results, assumptions, and algorithms. Our previous experience with using SOCR tools for educational purposes show that such hands-on interactive demonstrations, synergistic mathematical, computational and analytical demonstrations enhance student performance and understanding of underlying principles [13]. Thus, it is beneficial to combine a variety of different exploratory and explanatory techniques combined with visual representations within SOCRAT to extend its analytical capabilities.

Analytical tools design and development process is a basis for current migration of SOCR Modeler and Analyses into an application implemented in the SOCRAT environment. Modeler provides capabilities for visual fitting of various distributions to univariate data samples, for example, see Figure 8 for the custom implementation of the statistical t-test with power calculator, integrated with the interactive D^3^ chart. Analyses provides a number of tools for exploration of tabular data:

- statistical tests, such as t-test, ANOVA, etc.;
- power and sample size analysis;
- interactive clustering, including k-Means;
- dimensionality reduction with PCA, t-SNE, and other algorithms (Figures 7 and 11).

In addition we implement some problem-specific analytical solutions, such as an internal consistency calculator to evaluate instrument reliability from survey data, which is discussed in detail in the second case study below.

## 5. Example Case Studies

### 5.1. 3D cell morphological analysis

The first case study demonstrated visual analytics tools for exploratory analysis of three-dimensional (3D) morphological characteristics of cells in volumetric microscopic images. Cell morphology encapsulates various size and shape measures of cells and cell nuclei. Quantitative analysis of changes in size and shape of nuclear structures in 3D microscopic images is important for investigating basic nuclear organization and for detecting and treating pathological conditions such as cancer. In this case study, we use data derived from the 3D Cell Nuclear Morphology Microscopy Imaging Dataset [55]. This dataset includes a collection of volumetric microscopic images of human fibroblast cells in two phenotypic states: proliferating cells as untreated controls (PROLIF) and cells subjected to serum starvation (SS). It has been previously shown that serum starvation affects the size and shape of a cell nucleus, and thus should be reflected in morphological measures. Image analysis and feature extraction protocol included the following steps. First, binary masks of cell nuclei were obtained by the segmentation of 3D original data [55]. Then, a boundary surface of each cell nucleus binary mask was reconstructed from voxel data and discretized as a triangulated mesh [56,57]. Finally, following size and shape features were extracted from obtained surfaces:

- *class*: phenotypic state of the cell (PROLIF or SS);
- *Average Mean Curvature*: extrinsic measure of the surface curvature of the cell nucleus;
- *Compute Area*: surface area of the cell nucleus;
- *Curvedness*: magnitude of the local curvedness of the cell nucleus;
- *Fractal Dimension*: measure of the geometric complexity of the cell nucleus border;
- *Shape Index*: intrinsic measure of the relative curvature of the cell nucleus;
- *Volume*: volume of the cell nucleus.

The resulting dataset consisted of 934 rows each representing a single cell nucleus with 7 columns representing the features described above. We demonstrate the exploration of cell nuclear morphometry using interactive visual analytics tools available in SOCRAT.

**Figure 9.**
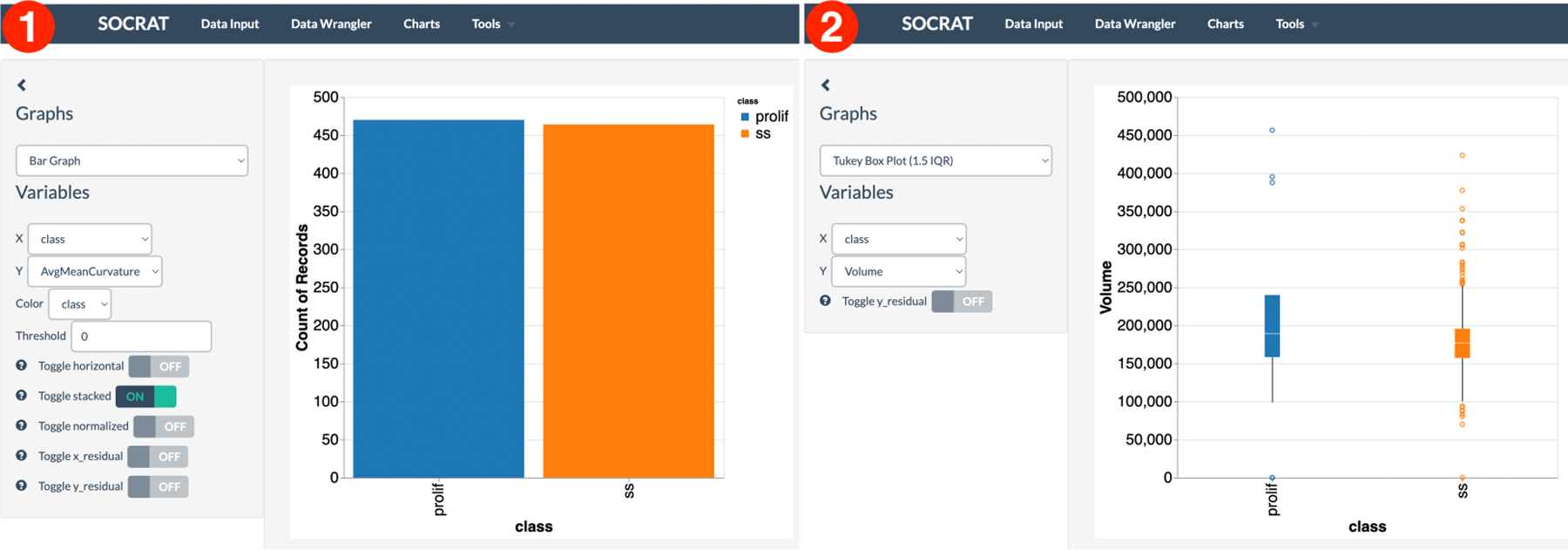
Interactive visualizations of cell morphology measures: **(1)** bar chart showing proportions of cells in the dataset per cell phenotypic class; **(2)** box plot showing the volume distributions of cell nuclei per treatment class.

First, the dataset is loaded at the Data Input tab in SOCRAT main menu by picking it from the list of available SOCR Datasets. Data grid shows the raw data values, column names, summary statistics, and histograms of value distributions per column, similar to those in Figure 3. For example, the *class* column contains two distinct values of the type “string”, which corresponds to correct cell phenotypic conditions (PROLIF and SS). Quick check in Data Wrangler confirms both the absence of missing values and consistency of data types. By clicking on the Filename column in Data Wrangler and selecting the first suggestion “Drop Filename”, we can remove it as filenames are not useful for the analysis. To explore single variable value distribution, a number of standard charts are available, with the support for interactive zoom, scaling, and highlighting. For example, checking the ratio of cells per phenotypic state can be quickly done using a simple bar chart that shows almost equal proportions, see Figure 9. A Tukey box plot can be then used to look at the distribution of each morphological measure across two cell classes, for example, cell nucleus volume (Figure 9).

**Figure 10.**
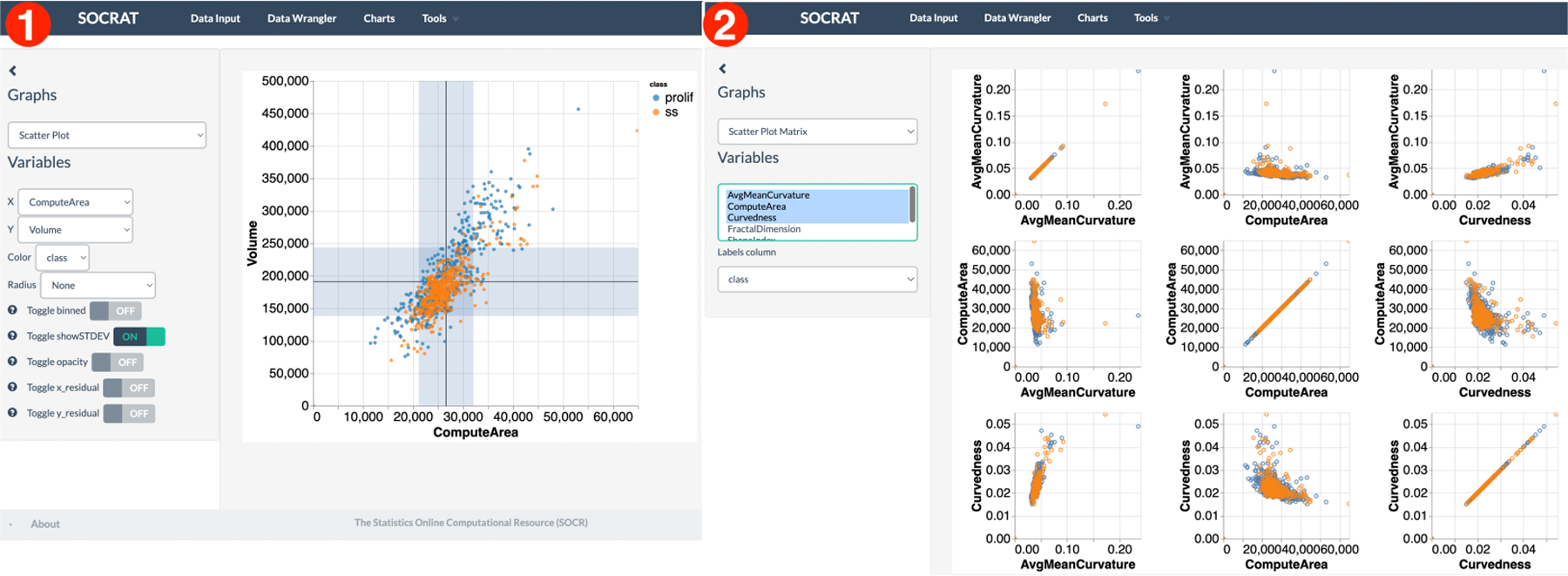
Multivariate interactive visualizations of cell morphology measures: **(1)** scatter plot demonstrating linear relationship between cell nucleus volume and surface area; **(2)** scatter plot matrix showing relationship between various nuclear morphology features.

Exploration of relationships between variables in the dataset is available through scatter plots and matrices, bubble plots, binned heatmaps, and other types of charts. For example, it would be reasonable to assume that volume and surface area of a cell nucleus should have a linear relationship. This assumption is confirmed clearly using a scatter plot in Figure 10. Horizontal and vertical bands show sample standard deviation for both variables. As shown in Figure 10, a scatter plot matrix can be used to find more interesting relationships between more than two measures. For example, it seems that serum-starved cells have nuclei with lower variability in curvedness, volume and shape index. They tend to be smaller in volume and have a lower shape index. These observations provide an insight in how these specifics changes in size and shape can be related to underlying biological mechanisms. Although these are these noticeable differences in value distributions across features, there is no clear separation between cell classes.

**Figure 11.**
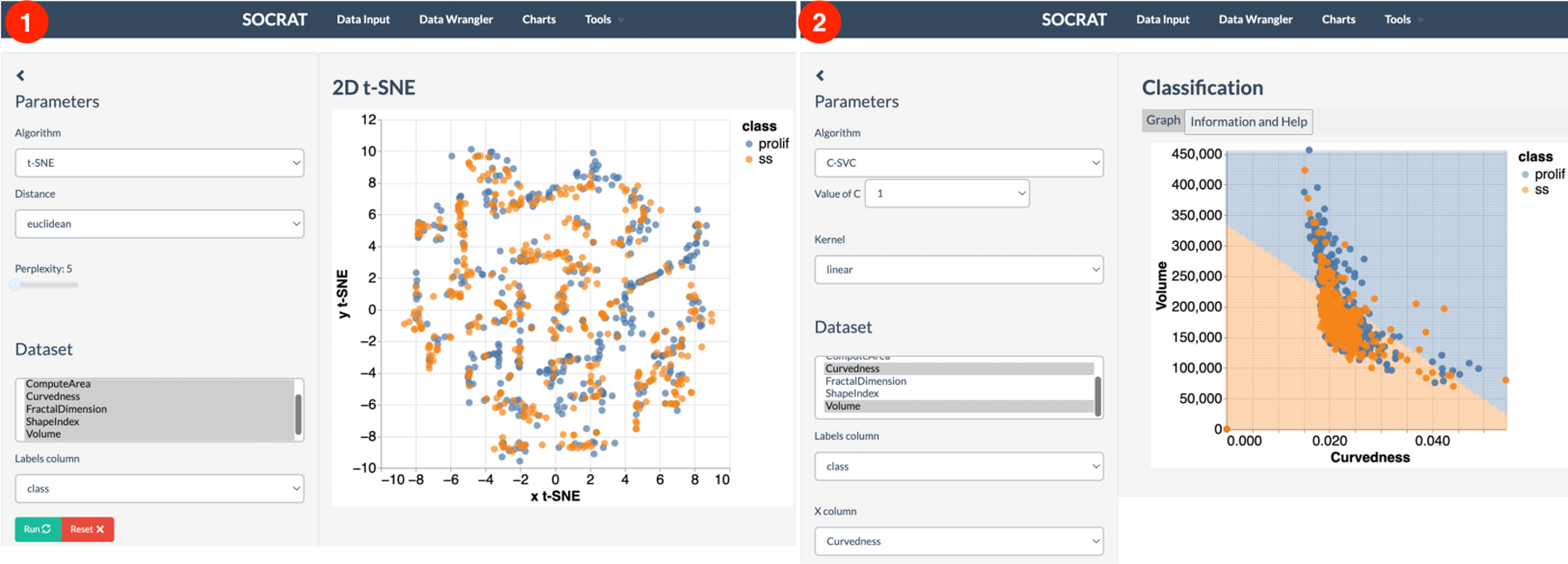
Interactive analysis of cell morphometry dataset: **(1)** unsupervised nonlinear dimensionality reduction using the t-SNE algorithm; **(2)** supervised classification using the SVM algorithm.

An alternative approach to explore the data may involve projecting it to a new space. SOCRAT implements the t-distributed Stochastic Neighbor Embedding (t-SNE) algorithm, a nonlinear dimensionality reduction technique well-suited for embedding high-dimensional data for visualization in a low-dimensional space [58]. As shown in Figure 11, t-SNE visualization shows the variability of data as the projection does not contain clearly separated clusters of cell classes and changing the perplexity (a t-SNE parameter) does not drastically affect the result. Further assessment of the possibility to discriminate between cell phenotypic states using nonlinear supervised classification algorithms, such as Support Vector Machines (SVM) [59], shows the model decision boundary that correctly captured most of the data points, but misclassified those outside of the distribution center, indicating that additional features may be required for better separation (Figure 11).

### 5.2. Internal consistency evaluation: Türkiye student evaluation data

Second case study focuses on development of a specific visual analytics module for evaluation of measurement reliability. Reliability is one of the core concepts in psychometric methodology. Reliable measures are consistently across time, individuals, and situations [60]. Internal consistency is typically a measure based on the correlations between different items on the same test and measures whether several items that propose to measure the same general construct and produce similar scores. Methods for evaluation of reliability and internal consistency methods are often used in health sciences. Development of a reliability evaluation tool based on SOCRAT aimed to support a training curriculum emphasizing the fundamentals, applications and practice of scientific methods for graduate students in the health sciences.

We focused on few measures of measurement reliability for this activity:

- Cronbach’s alpha is a coefficient of internal consistency that is commonly used as an estimate of the reliability;
- Intra-class correlation coefficient (ICC) assesses the consistency, or reproducibility, of quantitative measurements made by different observers measuring the same quantity. The ICC is defined as the ratio of between-cluster variance to total variance;
- Split-Half Reliability assessment, the test is split in half (e.g., odd / even) creating “equivalent forms”. The two “forms” are correlated with each other and the correlation coefficient is adjusted to reflect the entire test length, using the Spearman-Brown Prophecy formula.

Using integrated statistical and computational SOCRAT capabilities, we implemented a unique module to evaluate internal consistency by calculating Cronbach’s alpha , ICC, and Split-Half Reliability. Calculation of Cronbach’s alpha is supported by a number of various approaches to estimate confidence intervals [61], including bootstrap, Koning and Franses, logit, and novel asymptotically distribution-free [62] method to calculate confidence intervals.

As a test datasets for this case study we use Türkiye Student Evaluation Data [63]. Gazi University, Ankara, Turkey collected data consisting of 5,820 evaluation scores provided by Gazi University students. Each record consists of 5 meta-data attributes and 28 course specific questions (Q1-Q28). This study aims to identify the consistency of the evaluation instrument.

It included following variables:

- *Instr*: Instructor’s identifier with values taken from 1,2,3 ;
- *Class*: Course code (descriptor) with values taken from 1–13 ;
- *Repeat*: Number of times the student is taking this course with values taken from 0,1,2,3,...;
- *Attendance*: Code of the level of attendance with values from 0,1,2,3,4;
- *Difficulty*: Level of difficulty of the course as perceived by the student with values taken from 1,2,3,4,5;
- Q1-Q28: course specific questions measured in Likert scale 1,2,3,4,5.

**Figure 12.**
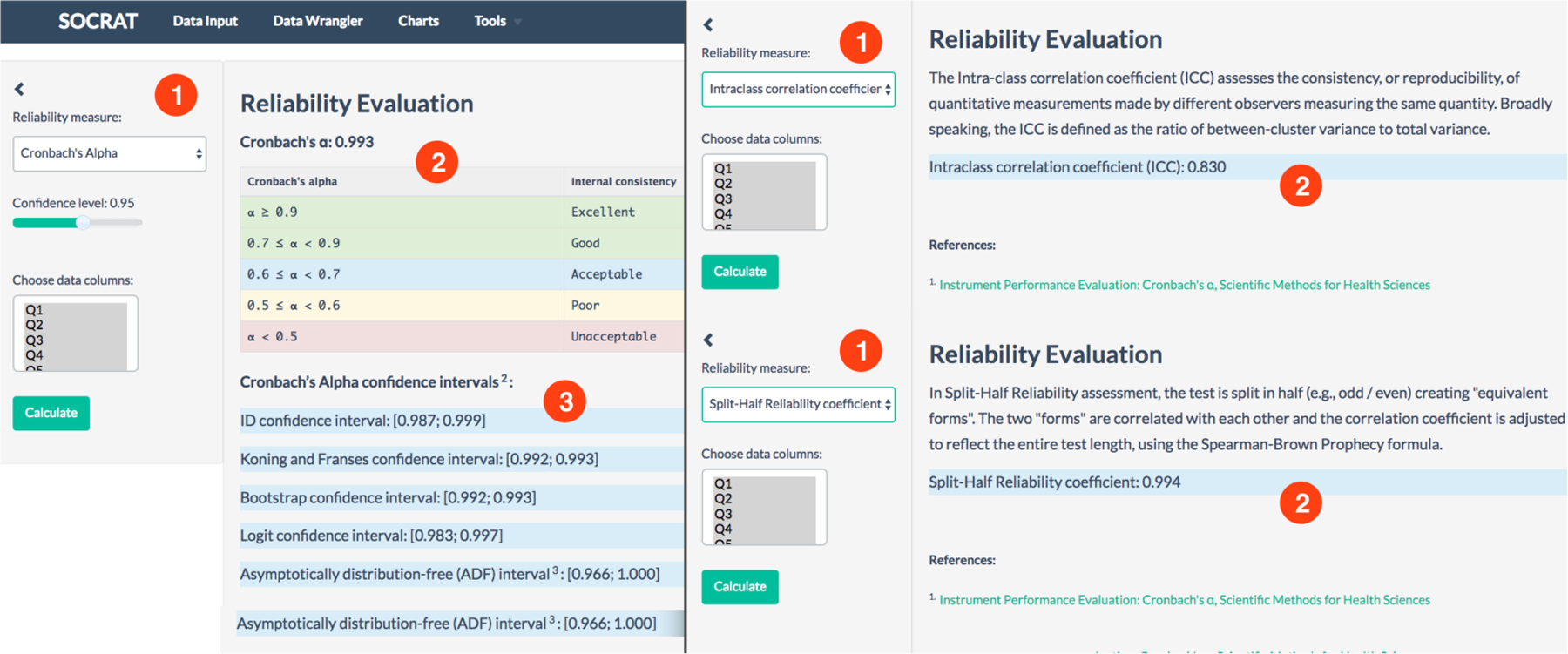
Reliability evaluation module: (1) Metric selection, (2) computed result and interpretation, **(3)** Cronbach’s alpha confidence intervals.

Before proceeding to reliability calculations, the user may choose only a few features, e.g., Q1-Q28, that contain course specific student responses. We can remove the first five columns of data using Data Wrangler, and then open Reliability evaluation. Cronbach’s alpha and its confidence intervals are evaluated automatically as the module starts. Results are reported together with general interpretation instructions, see Figure 12. On Türkeye Student Evaluation Data results for all three calculated metrics agreed and assessed reliability as high.

## 6. Discussion

In this paper, we presented Statistics Online Computational Resource Analytical Toolbox (SOCRAT), a dynamic, flexible, and extensible web-based visual analytics toolbox. SOCRAT implements a platform design based on multi-level modularity and declarative specifications that enables easy integration of a number of components for data management, analysis, and visualization. This allows SOCRAT to benefit from a diverse landscape of existing in-browser solutions by combining them with custom modules into a unique, powerful, feature-rich visual analytics toolbox. SOCRAT integrates a number of independently developed tools for data input, display, storage, interactive visualization, statistics and machine learning. Through use cases, we demonstrated how SOCRAT can be used for visual and statistical analysis of various datasets, providing a number of unique features.

In the future, we plan to extend SOCRAT with new features for data management, visualization, and analytics. Specifically, current version of SOCRAT only supports tabular data format and a few charts for nested JSON data structures. We plan to improve capabilities for working with hierarchical tabular data by using methods that enable identification of hierarchies in tabular data and provide transformations for aggregating and visualizing them [64]. As we continue implementing a wider variety of standard interactive charts, we recognize that SOCRAT still requires manual specifications for visual data exploration that can be tedious. To lower that barrier, we aim to employ data-driven visualisation recommendations [29] to automatically propose informative views of data to the user. Analytical capabilities of SOCRAT still do not match those of SOCR Analyses applets [31,32], therefore we plan to implement new techniques for statistical testing, regression modeling, and unsupervised learning, such as hierarchical clustering. Finally, performance of SOCRAT components, especially compute-heavy analysis tools, can be limited and cause delays, when dealing with large tabular data. When the loaded dataset contains over 20,000 entries, current version of SOCRAT displays a warning and suggests to uniformly subsample rows to reduce computational delays. However, uniform subsampling may be not optimal because it ignores internal structure of the dataset (i.e., how much information one observation carries). To address this issue, we will consider implementing other sampling procedures that better preserve important properties of data [65]. As subsampling leads to the loss of information that may be critical for the downstream analyses, we will further explore improving SOCRAT scalability to large tabular data by implementing analysis modules using WebAssembly [66], a binary-code executable format that can be run in modern web browsers at near-native speed. Previous studies reported WebAssembly outperforming native JavaScript in numerical computing tasks across different hardware platforms and browsers [6].

To enable open design validation and knowledge dissemination, SOCRAT is made publicly available as open-source software. SOCRAT users and other web-based VA tool developers are welcome to submit new features proposals and bug reports by creating a new GitHub issue in the SOCRAT repository. We also welcome new feature implementations, proposals to integrate specific third-party tools and other kinds of code contributions submitted as pull requests to the GitHub repository. More broadly, SOCRAT should be viewed as a proof-of-concept implementation of a modular platform architecture that allows combining various components into a customized web VA application. All data management, visualization and analytical components are optional and can be easily removed or substituted by alternatives to tailor SOCRAT functionality to a specific application. The GitHub repository provides installation instructions, such that it can be locally run, tested, modified and reconfigured according to individual preferences.

Overall, we hope that this work will engage the VA community into a discussion of architecture design practices for creating more effective and feature-rich VA solutions.

## Abbreviations

3D: Three-dimensional
AD: Alzheimer’s disease
ANOVA: Analysis of Variance
API: Application Programming Interface
CSV: Comma-separated Values
D^3^: Data-Driven Documents
EDA: Exploratory Data Analysis
HTML: HyperText Markup Language
ICC: Intra-class Correlation Coefficient
JSON: JavaScript Object Notation
MCI: mild cognitive impairment
MVC: Model-view-controller
NC: Normal controls
npm: Node Package Manager
PCA: Principal Component Analysis
PROLIF: Proliferating (cells)
SOCR: Statistics Online Computational Resource
SOCRAT: Statistics Online Computational Resource Analytical Toolbox
SS: Serum-starved (cells)
SVM: Support Vector Machine
t-SNE: t-distributed Stochastic Neighbor Embedding
TSV: Tab-separated Values
VA: Visual Analytics
UI: User Interface
URL: Uniform Resource Locator
